# Body mass index and measures of body fat for defining obesity and underweight: a cross-sectional, population-based study

**DOI:** 10.1186/2052-9538-1-9

**Published:** 2014-06-23

**Authors:** Julie A Pasco, Kara L Holloway, Amelia G Dobbins, Mark A Kotowicz, Lana J Williams, Sharon L Brennan

**Affiliations:** Epi-Centre for Healthy Ageing, School of Medicine, IMPACT SRC, Deakin University, PO Box 281, Geelong, Victoria 3220 Australia; NorthWest Academic Centre, The University of Melbourne, St Albans, Victoria Australia

**Keywords:** Body composition, Epidemiology, Obesity, Overweight, Percentage body fat, Prevalence, Underweight

## Abstract

**Background:**

The body mass index (BMI) is commonly used as a surrogate marker for adiposity. However, the BMI indicates weight-for-height without considering differences in body composition and the contribution of body fat to overall body weight.

The aim of this cross-sectional study was to identify sex-and-age-specific values for percentage body fat (%BF), measured using whole body dual energy x-ray absorptiometry (DXA), that correspond to BMI 18.5 kg/m^2^ (threshold for underweight), 25.0 kg/m^2^ (overweight) and 30.0 kg/m^2^ (obesity) and compare the prevalence of underweight, overweight and obesity in the adult white Australian population using these BMI thresholds and equivalent values for %BF. These analyses utilise data from randomly-selected men (n = 1446) and women (n = 1045), age 20–96 years, who had concurrent anthropometry and DXA assessments as part of the Geelong Osteoporosis Study, 2001–2008.

**Results:**

Values for %BF cut-points for underweight, overweight and obesity were predicted from sex, age and BMI. Using these cut-points, the age-standardised prevalence among men for underweight was 3.1% (95% CI 2.1, 4.1), overweight 40.4% (95% CI 37.7, 43.1) and obesity 24.7% (95% CI 22.2, 27.1); among women, prevalence for underweight was 3.8% (95% CI 2.6, 5.0), overweight 32.3% (95% CI 29.5, 35.2) and obesity 29.5% (95% CI 26.7, 32.3). Prevalence estimates using BMI criteria for men were: underweight 0.6% (95% CI 0.2, 1.1), overweight 45.5% (95% CI 42.7, 48.2) and obesity 19.7% (95% CI 17.5, 21.9); and for women, underweight 1.4% (95% CI 0.7, 2.0), overweight 30.3% (95% CI 27.5, 33.1) and obesity 28.2% (95% CI 25.4, 31.0).

**Conclusions:**

Utilising a single BMI threshold may underestimate the true extent of obesity in the white population, particularly among men. Similarly, the BMI underestimates the prevalence of underweight, suggesting that this body build is apparent in the population, albeit at a low prevalence. Optimal thresholds for defining underweight and obesity will ultimately depend on risk assessment for impaired health and early mortality.

## Background

Globally, obesity has nearly doubled over the last three decades [1]. In Australia, the prevalence of obesity among adults has increased from 11.1% in 1995 to 16.4% in 2004–05 [2] and this upward trend is predicted to continue over coming decades [3]. Global and local prevalence estimates are based on the body mass index (BMI) which provide a guide to obesity levels as recognised by BMI values greater than or equal to 30. Yet the BMI is a ratio of body weight-for-height [4] thereby limiting its usefulness as an indicator for adiposity because no account is made of variations in body composition. Clear shortcomings are evident when the BMI overestimates adiposity in muscular body builds and underestimates adiposity in the elderly [5, 6].

The simplicity and ease of measurement have entrenched the widespread use of the BMI as a marker of adiposity, not only for epidemiological purposes, but also in clinical practice. The aim of this study was to measure body fat mass using whole body dual energy x-ray absorptiometry (DXA) and BMI in a population-based sample of men and women in order to identify sex-and-age-specific values for percentage body fat (%BF) that correspond to internationally recognised BMI cut-points for defining underweight, overweight and obesity. This approach extends previous studies that utilised %BF thresholds of 25% for men and 35% for obesity [6, 7]. We have previously reported a temporal shift in the distribution of BMI in the population such that the prevalence of underweight women diminished between 1993–1997 and 2004–2007 [8], but that study did not investigate changes in body composition. The aim of this study was to compare prevalence estimates for underweight, overweight and obesity in the adult white Australian population using BMI thresholds for each category and the equivalent sex-and-age-specific cut-points for %BF.

## Methods

### Ethics statement

The study was approved by the Barwon Health Human Research Ethics Committee. All participants provided informed, written consent.

### Subjects

This cross-sectional study was conducted as part of the Geelong Osteoporosis Study (GOS), a population-based cohort study, set in the Barwon Statistical Division in south-eastern Australia [9]. Age-stratified samples of men and women were selected at random from the Commonwealth electoral roll, which provides an ideal sampling frame for epidemiological research in Australia because registration with the Australian Electoral Commission is compulsory for residents aged 18 years and over. In total, 1467 men were recruited 2001–2006 (67% participation, age 20–96 years) and 1494 women were recruited 1993–1997 (77% participation, age 20–93 years). This set of analyses utilises data collected at the baseline visit for 1467 men, and the 10-year follow-up (2003–2008) for 882 women (82% retention of eligible women). A further 194 women aged 20–29 years were recruited 2005–2008 (82% participation), providing a total sample 1076 women for this analysis. The cohort was essentially white; no indigenous Australians participated in the study. Details of participation and non-participation have been described elsewhere [9, 10].

### Body composition measures

Body weight was measured to ± 0.1 kg using electronic scales, standing height was measured to ± 0.001 m using a wall mounted stadiometer and BMI was calculated as weight/height^2^ (kg/m^2^). Based on WHO criteria [11], underweight was identified as BMI < 18.5 kg/m^2^, overweight as BMI 25.0-29.9 kg/m^2^, and obese as BMI ≥ 30.0 kg/m^2^. Measures of body fat mass, lean mass and bone mineral content were provided by whole body DXA using a Lunar DPX-L densitometer (software version 1.31; Lunar, Madison, WI, USA); however, 923 of the men were scanned on a GE-Lunar Prodigy (Prodigy; GE Lunar, Madison, WI, USA) when the DPX-L was decommissioned. No significant differences were detected in lumbar spine or femoral neck bone mineral density measurements when the scanners were cross calibrated on 40 subjects aged 21 to 82 years. The percentage body fat (%BF) was calculated as body fat mass expressed as a percentage of the sum of body fat mass, lean mass and bone mineral content. Individuals without valid whole body scans (21 men and 31 women) were excluded. Anthropometry was performed by trained personnel and the densitometer operators had completed the accredited Australian and New Zealand Bone and Mineral Society (ANZBMS) Clinical Densitometry Training Course and were licenced through the Department of Health State Government of Victoria to use radiation sources for research.

### Statistical analyses

The %BF values equivalent to the BMI cut-points 18.5, 25.0 and 30.0 kg/m^2^, which are used to identify underweight, overweight and obesity, respectively, were predicted using the following equation [5].


Variables include: sex (male = 1, female = 0), age (years) and BMI (kg/m^2^) centred around the mean (26.4 kg/m^2^) to reduce collinearity. The model includes interaction terms between sex and BMI, and sex and age. The equation had been derived previously using a subset of 1299 men and 855 women from the Geelong Osteoporosis Study for whom whole body DXA scans provided valid measures of body fat mass. Details of the development of this equation have been described elsewhere [5].

Individuals were classified as underweight, normal weight, overweight or obese according to published BMI cut-points and according to sex-and-age-specific %BF cut-points. A kappa (κ) statistic indicated the level of agreement between categories using the two sets of criteria. Sex-stratified prevalence estimates for underweight, overweight and obesity were determined according to BMI thresholds and the corresponding (calculated) %BF thresholds for age decades 20–79 years and 80 years and older, using mid-decade ages of 25, 35, 45, 55, 65, 75 and 85 years. Overall prevalence estimates were age-standardised to national age profiles using data from the Australian Bureau of Statistics (ABS cat. no. 2068.0 – 2006 Census Tables). A sensitivity analysis that compared prevalence estimates of obesity derived from BMI and %BF criteria was performed after excluding men scanned on the DPX-L densitometer. Statistical analyses were performed using Minitab (version 16, Minitab, State College, PA, USA).

## Results

### %BF thresholds for underweight, overweight and obesity

Mean predicted sex-and-age-specific %BF values that are equivalent to the BMI values of 18.5, 25.0 and 30.0 kg/m^2^ are shown in Table 1, where the data are stratified by sex and age-group. The %BF thresholds increased with age and were consistently lower for men than for women across all age-groups. The sex-and-age-specific thresholds for %BF were subsequently used to identify underweight, normal weight, overweight and obese individuals.

**Table 1:** **Mean sex-and-age-specific cut-points for percentage body fat equivalent to body mass index 18.5 kg/m** ^**2**^ **(underweight), 25.0 kg/m** ^**2**^ **(overweight) and BMI 30.0 kg/m** ^**2**^ **(obese)**

Age (yr)	Underweight		Overweight		Obese	
	Men	Women	Men	Women	Men	Women
20-29	9.3	21.8	20.6	36.0	27.5	43.4
30-39	9.9	22.0	21.2	36.2	28.1	43.6
40-49	10.5	22.2	21.8	36.4	28.7	43.8
50-59	11.1	22.4	22.4	36.6	29.3	44.0
60-69	11.7	22.6	23.0	36.8	29.9	44.2
70-79	12.3	22.8	23.6	37.0	30.5	44.4
80+	12.9	23.0	24.2	37.2	31.1	44.6

BMI body mass index (kg/m^2^); %BF percentage body fat mass.

### Prevalence of underweight, overweight and obesity

**Figure 1:**
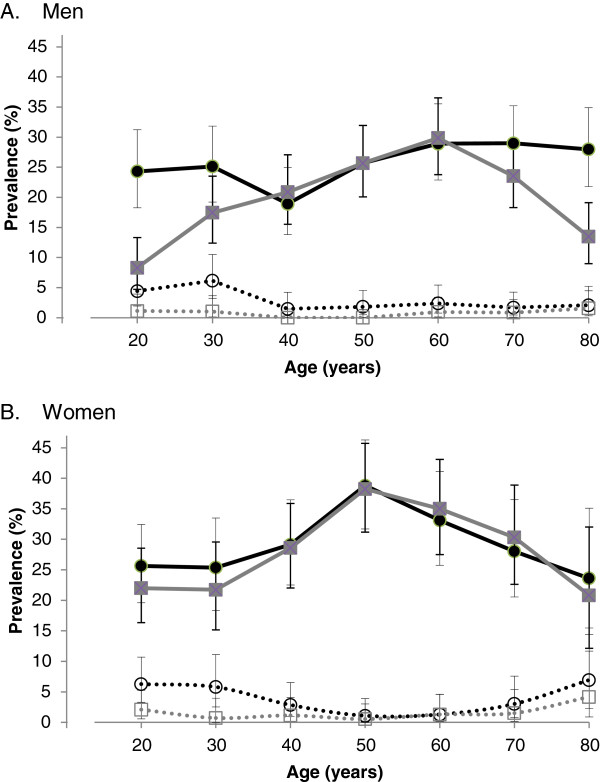
**Age-specific prevalence of obesity and underweight using body mass index and percentage body fat criteria.** Age-specific prevalence of obesity (solid lines and symbols) and underweight (broken lines and hollow symbols) defined using body mass index (BMI) thresholds (grey lines and square symbols) and sex-and-age-specific percentage body fat (%BF) thresholds (black lines and circular symbols). Data are for **(A)** men and **(B)** women by age decades (20 represents 20–29 years, etc.). Data are shown as mean and 95% confidence intervals.

Numbers of men and women in the categories of underweight, ideal weight, overweight and obese by age-group are shown in Table 2. Mean BMI and %BF values for men and women by age-group are listed in Table 3. Mean prevalence estimates for underweight, overweight and obesity according to BMI thresholds and sex-and-age-specific %BF thresholds, for men and women stratified by age-group, are shown in Table 4 and presented graphically for underweight and obesity in Figure 1. In women, both methods indicated that the prevalence of obesity increased with age until 50–59 years, followed by an age-related decline. The age-related profile for men according to BMI criteria showed an age-related increase that peaked at age 60–69 years followed by an age-related decline; however, %BF values indicate that obesity was under-estimated in younger men and elderly men than BMI would suggest. For both sexes, the prevalence of overweight was similar for both BMI and %BF criteria. The BMI tended to under-estimate the prevalence of underweight in both sexes particularly for young adults.

**Table 2:** **Numbers of individuals in each BMI category (underweight, ideal weight, overweight and obese) stratified by sex and age**

Age (yr)	Underweight		Ideal weight		Overweight		Obese	
	Men	Women	Men	Women	Men	Women	Men	Women
20-29	2	4	111	103	53	42	15	42
30-39	2	1	76	73	83	34	34	30
40-49	0	2	61	63	102	60	43	50
50-59	0	1	42	54	123	58	57	70
60-69	2	2	43	46	103	52	63	54
70-79	2	2	59	37	121	53	56	40
80+	3	3	67	27	97	27	26	15
All	11	15	459	403	682	326	294	301

BMI body mass index (kg/m^2^).

BMI categories: Underweight BMI < 18.5 kg/m^2^, ideal weight BMI 18.5-24.9 kg/m^2^, overweight BMI 25.0-29.9 kg/m^2^ and obese BMI ≥ 30.0 kg/m^2^.

**Table 3:** **Mean (standard deviation) values for body mass index and percentage body fat stratified by sex and age**

	Men		Women	
Age (yr)	BMI	%BF	BMI	%BF
20-29	24.6 (3.5)	20.8 (8.1)	26.2 (6.5)	36.1 (9.5)
30-39	26.3 (3.7)	22.8 (7.1)	26.5 (5.9)	36.7 (9.2)
40-49	27.3 (3.9)	24.2 (5.5)	27.7 (6.0)	38.7 (8.1)
50-59	28.0 (4.0)	25.8 (6.2)	29.0 (6.1)	41.3 (8.3)
60-69	27.2 (4.2)	26.7 (6.4)	28.0 (5.1)	40.5 (7.4)
70-79	27.3 (4.0)	26.9 (6.4)	27.9 (4.9)	40.9 (7.6)
80+	26.1 (3.3)	27.1 (6.9)	26.4 (4.4)	38.4 (7.9)

BMI body mass index (kg/m^2^); %BF percentage body fat mass.

**Table 4:** **Sex-and-age-stratified prevalence (mean percentage and 95% confidence interval) of underweight, overweight and obesity determined by body mass index and sex-and-age-specific percentage body fat mass criteria**

		Age (yr)	20-29	30-39	40-49	50-59	60-69	70-79	80+
Men		n	181	195	206	222	211	238	193
	BMI	Underweight	1.1 (0.1, 3.9)	1.0 (0.1, 3.7)	0.0 (0.0, 1.4)	0.0 (0.0, 1.3)	0.9 (0.1, 3.4)	0.8 (0.1, 3.0)	1.6 (0.3, 4.5)
		Overweight	29.3 (22.8, 36.5)	42.6 (35.5, 49.8)	49.5 (42.5, 56.4)	55.4 (48.6, 62.1)	48.8 (41.9, 55.8)	50.8 (44.3, 57.4)	50.3 (43.0, 57.5)
		Obese	8.3 (4.7, 13.3)	17.4 (12.4, 23.5)	20.9 (15.5, 27.1)	25.7 (20.1, 31.9)	29.9 (23.8, 36.5)	23.5 (18.3, 29.4)	13.5 (9.0, 19.1)
	%BF	Underweight	4.4 (1.9, 8.5)	6.2 (3.2, 10.5)	1.5 (0.3, 4.2)	1.8 (0.5, 4.5)	2.4 (0.8, 5.4)	1.7 (0.5, 4.2)	2.1 (0.6, 5.2)
		Overweight	22.7 (16.8, 29.4)	34.9 (28.2, 42.0)	51.5 (44.4, 58.5)	49.5 (42.8, 56.3)	42.7 (35.9, 49.6)	44.1 (37.7, 50.7)	42.0 (34.9, 49.3)
		Obese	24.3 (18.3, 31.2)	25.1 (19.2, 31.8)	18.9 (13.8, 25.0)	25.7 (20.1, 31.9)	28.9 (22.9, 35.5)	29.0 (23.3, 35.2)	28.0 (21.8, 34.9)
**Women**		**n**	**191**	**138**	**175**	**183**	**154**	**132**	**72**
	BMI	Underweight	2.1 (0.6, 5.3)	0.7 (0.0, 4.0)	1.1 (0.1, 4.1)	0.5 (0.0, 3.0)	1.3 (0.2, 4.6)	1.5 (0.2, 5.4)	4.2 (0.9, 11.7)
		Overweight	22.0 (16.3, 28.5)	24.6 (17.7, 32.7)	34.3 (27.3, 41.8)	31.7 (25.0, 39.0)	33.8 (26.4, 41.8)	40.2 (31.7, 49.0)	37.5 (26.4, 49.7)
		Obese	22.0 (16.3, 28.5)	21.7 (15.2, 29.6)	28.6 (22.0, 35.9)	38.3 (31.2, 45.7)	35.1 (27.6, 43.2)	30.3 (22.6, 38.9)	20.8 (12.2, 32.0)
	%BF	Underweight	6.3 (3.3, 10.7)	5.8 (2.5, 11.1)	2.9 (0.9, 6.5)	1.1 (0.1, 3.9)	1.3 (0.2, 4.6)	3.0 (0.8, 7.6)	6.9 (2.3, 15.5)
		Overweight	23.6 (17.7, 30.2)	25.4 (18.3, 33.5)	34.9 (27.8, 41.4)	35.0 (28.1, 42.4)	34.4 (27.0, 42.5)	53.0 (44.2, 61.8)	36.1 (25.1, 48.3)
		Obese	25.7 (19.6, 32.5)	25.4 (18.3, 33.5)	29.1 (22.5, 36.5)	38.8 (31.7, 46.3)	33.1 (25.8, 41.1)	28.0 (20.6, 36.5)	23.6 (14.4, 35.1)

BMI body mass index (kg/m^2^); %BF percentage body fat mass.

A sensitivity analysis that excluded men scanned on the DPX-L densitometer showed that the age-related patterns based on %BF criteria were sustained and, importantly, significant differences persisted between prevalence estimates based on BMI and %BF criteria for the two age groups 20–29 years and 80+ years obesity: prevalence for age 20–29 years BMI 8.2% (95% CI 4.5, 13.4) and %BF 25.1% (95% CI 18.8, 32.3), and for age 80+ BMI 13.4% (95% CI 8.7, 19.5) and %BF 29.8% (95% CI 23.1, 37.3).

According to age-specific %BF criteria for men, the overall mean age-standardised prevalence for underweight was 3.1% (95% CI 2.1, 4.1), overweight 40.4% (95% CI 37.7, 43.1) and obesity 24.7% (95% CI 22.2, 27.1). For women, the prevalence for underweight was 3.8% (95% CI 2.6, 5.0), overweight 32.3% (95% CI 29.5, 35.2) and obesity 29.5% (95% CI 26.7, 32.3).

According to BMI criteria for men, the overall mean age-standardised prevalence for underweight was 0.6% (95% CI 0.2, 1.1), overweight 45.5% (95% CI 42.7, 48.2) and obesity 19.7% (95% CI 17.5, 21.9). For women, the prevalence for underweight was 1.4% (95% CI 0.7, 2.0), overweight 30.3% (95% CI 27.5, 33.1) and obesity 28.2% (95% CI 25.4, 31.0). Thus, the mean age-standardised prevalence for underweight for both men and women was lower according to BMI. For men, the age-standardised prevalence for obesity was similarly lower according to BMI; for women the difference in estimates of age-standardised prevalence for obesity based on BMI and %BF was not significant. No differences were detected in age-standardised prevalence estimates for overweight in either sex.

### Agreement between categories based on BMI and %BF criteria

There was exact agreement using sex-and-age-specific %BF and BMI criteria for categorising underweight, ideal weight, overweight and obese groups for 62.6% men (κ = 0.4) and 73.9% women (κ = 0.6); agreement to within one category was observed for 98.7% men and 99.8% women. Whereas 82.7% of women classed as obese according to BMI were also identified as obese according to sex-and-age-specific %BF criteria, only 68.4% of men classed as obese by BMI were similarly classified by sex-and-age-specific %BF. On the other hand, 80.1% of women and 53.9% of men who were identified as obese according to sex-and-age-specific %BF criteria, had BMI ≥ 30.0 k/m^2^.

## Discussion

Using sex-and-age-specific cut-points for %BF equivalent to BMI 30.0 kg/m^2^, we report that 24.7% (95% CI 22.2, 27.1) of men and 29.5% (95% CI 26.7, 32.3) of women were obese. The prevalence estimate for men was greater than the estimate of 19.7% (95% CI 17.5, 21.9), which was based on BMI criteria. The pattern was similar for women for whom the prevalence of obesity according to the BMI was 28.2% (95% CI 25.4, 31.0); however, the difference in the estimates was not significant. For both sexes, the prevalence of underweight was lower according to BMI. Whereas three-quarters of the women were similarly classified into groupings ranging from underweight to obese according to both %BF and BMI criteria, exact agreement was observed for less than two-thirds of the men.

Our approach was similar to that reported by Gallagher et al. [12] who derived %BF cut-points from several diagnostic techniques, including DXA, which corresponded to the published BMI thresholds for underweight, overweight and obesity. Prediction equations for %BF were evaluated for adults (from BMI, sex, age and ethnicity) in order to identify healthy %BF ranges. Similar techniques have been employed by others in order to evaluate the validity of the BMI threshold for obesity in different populations [6, 7, 13, 14]. However, to our knowledge, no other study has utilised sex-and-age-specific %BF thresholds equivalent to published BMI thresholds to compare prevalence estimates of underweight, overweight and obesity.

Our results suggest that 17.3% of women and 31.6% of men who were identified as obese according to BMI were misclassified according to sex-and-age-specific %BF criteria. The inability to distinguish the different contributions to body weight, of fat and non-fat tissue (such as muscle and bone, which have greater densities than fat), explains why the BMI might overestimate adiposity in muscular and lean body builds. On the other hand, only 80.1% of women and 53.9% of men in our study who were classified as obese using sex-and-age-specific %BF thresholds had BMI in the obese range. As a corollary, 19.9% of women and 46.1% men with high %BF were overlooked as being obese according to BMI criteria. The BMI might underestimate adiposity as a consequence of age-related lean tissue loss, particularly skeletal muscle, and accumulation of fat; these are characteristics of sarcopenic obesity seen in the elderly [15, 16]. Results from our study support the contention that BMI underestimates adiposity in elderly men (aged 70 years and older). Paradoxically, our study also suggests that the BMI markedly underestimated adiposity in young men (aged 20–29 years). It seems likely that for this group, body fat contributes more, and lean tissue less, to body weight than in other groups. While the reasons for this remain unclear, we might speculate that the fat-to-lean tissue mass ratio is disproportionately high as a result of unhealthy lifestyle choices including sedentary behaviour and poor nutrition. Differences in body composition might also be related to an increasing prevalence of growth hormone deficiency with increasing age, resulting in loss of lean tissue and increases in body fat [17]. These findings have public health implications, as the prevalence of adult obesity as described by the BMI, may be underestimated at a population level, particularly among men.

Both the BMI and %BF identify weight or fat mass relative to the whole body, but this has been conceptualized differently for the two indices. The BMI expresses body weight (kg) relative to stature (height, m^2^) and it should be noted that adjustment for height in this index is suboptimal [18]. The second order polynomial relationship between BMI and %BF [5] is partly explained by the relative relationship of body fat mass to total body weight; increments in body fat mass result in diminishing increments in %BF. Furthermore, accumulation of body fat in healthy bodies is generally accompanied by a compensatory response from the musculoskeletal system, acting through mechanoreceptors in muscle and bone, as it adapts to better cope with the increasing mechanical load [19]. Adipokines also act as regulatory messengers between adipocytes in fat deposits, muscle [20] and bone [21, 22]. However, with excessive accumulation of body fat, the increased loading could exceed compensatory musculoskeletal responses thereby altering the proportions of fat, lean and bone issue. As a consequence, increases in BMI could reflect increased weight-for-height yet mask changes in body composition. Considering the obesity epidemic, a more accurate indicator of body fatness is required to better assess obesity-related health risks.

Our study has several strengths and limitations. The major strength is that study participants were selected at random from a clearly-defined population and this is important when reporting prevalence estimates. Furthermore, body composition was measured using anthropometric values (weight and height) in addition to whole body densitometry which provided a more accurate assessment of body fat mass. In the absence of cross-calibration data between the two densitometers, a sensitivity analysis that restricted comparisons for men scanned on one densitometer alone showed similar patterns to the full dataset. However, we cannot exclude the possibility of differences between the two machines. We acknowledge, however, that DXA measurements may be obscured by increasing levels of body fat. Lastly, our data relate to an essentially white population and the findings may not be pertinent to other ethnicities.

## Conclusions

We report that the prevalence of obesity using a BMI threshold may underestimate the true extent of obesity in the white population, particularly among young and elderly men. We also report that for both sexes, the prevalence of underweight using a BMI threshold may underestimate the true extent in the population. We suggest that optimal sex-and-age-specific thresholds be implemented for defining underweight and obesity in terms of body fat and recognise that such definitions will depend on risk assessment for disease, morbidity and mortality.
